# The first biological choice in patients with rheumatoid arthritis: data from the Moroccan register of biotherapies

**DOI:** 10.11604/pamj.2021.38.183.27081

**Published:** 2021-02-17

**Authors:** Meryem Eddaoudi, Samira Rostom, Ihsane Hmamouchi, Imane El Binoune, Bouchra Amine, Redouane Abouqal, Lahsen Achemlal, Fadoua Allali, Imane El Bouchti, Abdellah El Maghraoui, Imad Ghozlani, Hasna Hassikou, Taoufik Harzy, Linda Ichchou, Ouafae Mkinsi, Redouane Niamane, Rachid Bahiri

**Affiliations:** 1 Rheumatology Department A, El Ayachi Hospital, Ibn Sina University Hospital, Rabat-Sale, Morocco,; 2 Laboratory of Epidemiology and Clinical Research, Faculty of Medicine and Pharmacy, Rabat, Morocco,; 3 Provincial Hospital, Skhirat-Temara, Morocco,; 4 Rheumatology Department, Military Hospital Mohamed V, Ibn Sina University Hospital, Rabat, Morocco,; 5 Rheumatology Department B, El Ayachi Hospital, Ibn Sina University Hospital, Rabat-Sale, Morocco,; 6 Rheumatology Department, Arrazi Hospital, University Hospital Mohammed VI, Marrakech, Morocco,; 7 Rheumatology Medical Office, Rabat, Morocco,; 8 Rheumatology Department, University Hospital, Agadir, Morocco,; 9 Rheumatology Department, Military Hospital, Hassan II University Hospital, Meknes, Morocco,; 10 Rheumatology Department, University Hospital Hassan II, Fez, Morocco,; 11 Rheumatology Department, University Hospital Mohammed VI, Oujda, Morocco,; 12 Rheumatology Department, University Hospital Ibn Rochd, Casablanca, Morocco,; 13 Rheumatology Department, Military Hospital Avicenne, University Hospital Mohammed VI, Marrakech, Morocco

**Keywords:** Rituximab, rheumatoid arthritis, treatment, tumor necrosis factor inhibitors, tocilizumab

## Abstract

**Introduction:**

the aim of our study is to determine, from data of the Moroccan register of biotherapies, the factors influencing the choice of the first prescribed biological treatment.

**Methods:**

cross-sectional multicenter study including rheumatoid arthritis patients who were initiated the first biological treatment either: Rituximab, an anti-TNF, or Tocilizumab. The determinants related to the patient and disease have been gathered. A univariate and then multivariate analysis to determine the factors associated with the choice of the first bDMARDs was realized.

**Results:**

a total of 225 rheumatoid arthritis patients were included in the Moroccan registry. The mean age was 52 ± 11 years, with female predominance 88% (n = 197). The first prescribed biological treatment was Rituximab 74% (n = 166), the second one was Tocilizumab, 13.6% (n = 31) then comes the anti-TNF in 3^rd^ position with 12.4% (n = 28). The factors associated with the choice of Rituximab as the first line bDMARDs prescribed in univariate analysis were: the insurance type, the positivity of the rheumatoid factor. In multivariate analysis, only the insurance type that remains associated with the choice of Rituximab as the first biological drugs. The Tocilizumab was associated with shorter disease duration and was more prescribed as mono-therapy compared to non Tocilizumab group. TNFi was associated with the insurance type.

**Conclusion:**

our study suggests that Rituximab and TNFi are associated with the type of insurance and Tocilizumab is the most prescribed biologic mono-therapy in RA patients. Further studies are needed to confirm these results.

## Introduction

Rheumatoid arthritis (RA) is a chronic inflammatory disease of unknown origin affecting nearly 0.5 to 1% of the general population [1-3], characterized by chronic inflammation of small and large joints with structural progression and increasing functional disability over time [1, 4]. The management of RA is based on early diagnosis and treatment using a treat-to-target approach based on the assessment of disease activity [1, 5]. At the early stage of treatment for rheumatoid arthritis, the conventional Modifying Anti-Rheumatic Drugs (cDMARDs) are used in mono-therapy or in combination, namely Methotrexate, Sulfasalazine, Leflunomide and Antimalarial. In case cDMARDs failure or when these substances are to be discontinued due to the appearance of side effects, biological therapy is used [6].

Over the last three decades, there has been a rapid development of the biological disease-modifying anti-rheumatic drugs (bDMARDs) that are currently available for the treatment of RA in order to achieve reduced disease activity and clinical remission [7]. The bDMARDs are characterized by different mechanisms of action depending on the potential targets involved in the pathogenesis of the disease [1]. Tumour necrosis factor inhibitors (TNFis) were the first biological treatment developed for RA and most prescribed in recent decades for patients who failed treatment with cDMARDs [1], while Rituximab has been indicated as a second-line therapy after the failure of the first biological treatment [8].

Studies have shown that there is no big difference between the different classes of bDMARD in terms of clinical, functional and radiographic efficacy [9, 10]; with a unique exception of tocilizumab monotherapy [11], and in some circumstances Rituximab is indicated as the first bDMARD in case of history of lymphoma or demyelinating disease [12]. In our Moroccan context, the indication of Rituximab is influenced by the medico-economic factor of biological treatments as well as the risk of tuberculosis given the endemic situation of this pathology in our country. The best approach for each patient is still little known despite the broad and evolving spectrum of different bDMARDs currently available. Thus, the choice of the first biological drug is left to the practitioner´s choice and his/her personal experiences [13].

In the literature, several factors have been advocated as parameters of choice of the first bDMARDs such as comorbidities, cardiovascular risk, infectious risk, preference for a specific route of administration, predictive biomarkers such as seropositivity for rheumatoid factor (RF) and/or anti-citrullinated protein antibodies (ACPA), the cost-effectiveness and type of health coverage [4, 8, 13]. The aim of our study is to determine, from the data of the Moroccan register of biotherapies (RBSMR), the factors influencing the choice of the first prescribed bDMARDS.

## Methods

Patients enrolled in the Moroccan Register of Biotherapies (RBSMR) including patients undergoing rheumatoid arthritis and having gone through a biological treatment either Rituximab, Anti-TNF (TNFi) or Tocilizumab (TCZ) as the first bDMARD, the details of the data collected have already been published [14].

Socio-demographic and economic data pertaining to the patient including: age, sex, type of health insurance (mandatory health insurance (CNOPS, CNSS, FAR)), private insurance and RAMED (medical assistance scheme for people in economic decline) as well as factors related to the disease including: sedimentation rate (ESR), protein-C-reactive (CRP), Disease Activity Score (DAS28-ESR), rheumatoid factor (RF) and anti-citrullinated peptide antibody (ACPA) status, erosion presence, disease duration, diagnostic delay and lag time of Methotrexate initiation were collected.

**Statistical analysis:** in descriptive statistics, more continuous data was presented as mean and standard deviation (SD) or as median and inter-quartile range (IQR) according to the distribution, and categorical data were summarized as a frequency and in percentage. The first bDMARDs prescribed in the Moroccan register of biotherapy was identified. We performed a univariate analysis using a comparison test and a multivariate analysis to determine the factors associated with the choice of the first prescribed biological treatment. We have chosen the threshold element of significance P <0.05.

**Ethics approval and consent to participate:** the protocol for the original RBSMR study was reviewed and approved by local institutional review boards and the national ethic committee: Ethics committee for biomedical research Mohammed V university- RABAT Faculty of medicine and pharmacy of RABAT. The committee´s reference number: 117/17.

## Results

A total of 225 patients were included. The mean age of our patients was 52 ± 11.36 years with a female predominance N = 197 (87.60%). Most patients had severe activity with a mean DAS28 5.2 ± 1. Ninety percent of our population was seropositive. Regarding the choice of the first bDMARDs prescribed, Rituximab ranks first with 74%, followed by TCZ (13.6%) and TNFi comes last with 12,4% (Table 1).

**Table 1:** characteristics of PR patients from the RBSMR register

Features	N: 225
Age ¹ (years)	51 ± 11.36
Female sex ^2^	197 (87.60)
**Type of health insurance**	
RAMED^2^	97 (43.1)
CNSS^2^	5 (2.2)
CNOPS^2^	43 (19.1)
FAR^2^	74 (32.9)
Private insurance^2^	2 (0.9)
Other	1 (0.4)
DAS28 ¹	5.2 ± 1
Positive rheumatoid factor * ^2^	192 (90.5)
Positive ACPA* ^2^	151 (88.8)
**First bDMARS prescribes**	
* Rituximab ^2^	166 (74)
* Tocilizumab ^2^	31 (13.6)
* Anti-TNF alpha ^2^	28 (12.4)

1: Mean and standard deviation, 2: Number and percentage, 3: Inter-quartile median, * Data available ACPA: anti-citrullinated protein antibodies. RAMED (Régime d'Assistance Médicale aux économiquement démunis): health insurance for poor and vulnerable population. CNSS (Caisse Nationale de Sécurité Sociale): insurance for private sector employees. CNOPS (Caisse Nationale des Organismes de Prévoyance Sociale): insurance for public sector employees. FAR (assurance forces armées royales): insurance for military employees.

Rituximab was associated with the concomitant intake of cDMARDS compared to the non-Rituximab group (TCZ and TNFi) with a statistically significant p (p <0.0001) as well as the type of insurance RAMED (p = 0.003) and the rheumatoid factor (p = 0.05) (Table 2).

**Table 2:** comparison of features between the groups rituximab/non-rituximab

Features	Rituximab group	Non - Rituximab group	p
Age ¹ (years)	51 ± 11	51 ± 11	0.2
Female sex ^2^	146 (73.8)	51 (86.4)	0.7
**Type of health insurance**			
RAMED^2^	81 (49.7)	16 (27.1)	0.003
CNSS^2^	3(1.8)	2 (3.4)	0.4
CNOPS^2^	31 (19)	12 (20.3)	0.8
FAR^2^	46 (28.2)	28 (47.5)	0.07
Private insurance^2^	2 (1.2)	-	1
Duration of evolution ¹ (months)	236 ± 132	169 ± 54	0.08
Diagnosis lag time^3^ (months)	12 (1 - 48)	14 (1 - 60)	0.6
Initiation Methotrexate lag time^3^ (months)	18 (0.05 - 51)	42 (4 - 83)	0.1
Sedimentation rate ¹ (mm/1st hour)	42.05± 24.3	41.98 ± 26.31	0.9
C-reactive protein^3^ (mg/l)	24.50 (11.25 - 48.75)	14.50 (5.75 - 31.25)	0.07
DAS28 ¹	5.2 ± 1	5.2 ± 1	0.8
Positive rheumatoid factor * ^2^	144 (92.9)	48 (84.2)	0.05
Positive ACPA* ^2^	115 (89.8)	36 (85.7)	0.4
Carpite/erosions ^2^	82 (49.4)	29 (49.2)	0.9
Concomitant cDMARDs^2^	161 (97)	46 (78)	<0.0001
Arterial hypertension^2^	24 (14.5)	13 (22)	0.09
Diabetes^2^	34 (20.5)	12 (20.3)	0.2
Hypercholesterolemia^2^	18 (10.8)	10 (17.2)	0.2

1: Mean and standard deviation, 2: Number and percentage, 3: Inter-quartile median, * Data available ACPA : anti-citrullinated protein antibodies, RAMED (Régime d'Assistance Médicale aux économiquement démunis): health insurance for poor and vulnerable population, CNSS (Caisse Nationale de Sécurité Sociale): insurance for private sector employees, CNOPS (Caisse Nationale des Organismes de Prévoyance Sociale): insurance for public sector employees, FAR (assurance forces armées royales): insurance for military employees.

TCZ was associated with a shorter disease duration (150.33 ± 15.01 months) compared to the non-TCZ group (Rituximab and TNFi) (235.5 ± 126.49 months); p = 0.04. TCZ was significantly more prescribed as mono-therapy compared to non-TCZ group (p < 0, 0001). Regarding comorbidities, arterial hypertension was more presented in the TCZ group compared to the non-TCZ group (p = 0.03) and conversely for diabetes which was more associated with the non-TCZ group (p = 0.02). There was actually no big difference among the two groups regarding: age, sex, type of health insurance, delay diagnosis, RF and ACPA status, erosion presence and disease activity (Table 3).

**Table 3:** comparison of features between the groups TCZ/Non-TCZ

Features	TCZ group	Non-TCZ group	p
Age ¹ (years)	51.29 ± 10.78	52.04 ± 11.47	0.2
Female sex ^2^	28 (90.3)	169 (87.1)	0.6
**Type of health insurance**			
RAMED^2^	12 (38.7)	85 (43.8)	0.5
CNSS^2^	1(3.2)	4 (2.1)	0.6
CNOPS^2^	5 (16.1)	38 (19.6)	0.6
FAR^2^	13 (41.9)	61 (31.4)	0.2
Private insurance^2^		2 (1)	0.5
Duration of evolution ¹ (months)	150.33 ± 15.01	235.5 ± 126.49	0.04
Diagnosis lag time^3^ (months)	18 (1-49.5)	12 (1 - 48)	0.6
Initiation methotrexate lag time^3^ (months)	53.9 (4.4-110.4)	23.9(0.13-71.29)	0.3
Sedimentation rate ¹ (mm/1^st^ hour)	42.50 ± 25.77	41.95 ± 24.69	0.7
C-reactive protein^3^ (mg/l)	9.5 (4.5-49)	23.50 (9-42.50)	0.1
DAS28 ¹	5.1 ± 1	5.2 ± 1	0.5
Positive rheumatoid factor * ^2^	25 (80.6)	167 (86.1)	0.1
Positive ACPA* ^2^	19 (61.3)	132 (68)	0.6
Carpite / erosions ^2^	15 (48 .4)	96 (49.5)	0.4
Concomitant cDMARDs^2^	21 (67.7)	186 (95.9)	<0.0001
Arterial hypertension^2^	6 (19.4)	31 (16)	0.03
Diabetes^2^	4 (12.9)	42 (21.6)	0.02
Hypercholesterolemia^2^	5 (16.7)	23 (11.9)	0.4

1: Mean and standard deviation, 2: Number and percentage, 3: Inter-quartile media, * Data available

Comparing the TNFi and non-TNFi groups (Ritux and TCZ), the type of insurance FAR was more recorded in the TNFi group 15 (53.6%) patients compared to the non-TNFi group 59 (29.9%) patients; p = 0.01, contrary to the type of insurance RAMED which was more often reported in the non-TNFi group 93 (47.2%) patients compared to the TNFi group 4 (14.3%) patients; (P = 0.001) (Table 4).

**Table 4:** comparison of features between the groups TNFi/Non-TNFi

Features	TNFi group	Non-TNFi group	p
Age ¹ (years)	52.64±12.05	51.84±11.29	0.6
Female sex ^2^	23 (82.1)	174 (88.3)	0.3
**Type of health insurance**			
RAMED^2^	4 (14.3)	93 (47.2)	0.001
CNSS^2^	1(3.6)	4 (2)	0.6
CNOPS^2^	7 (25)	36 (18.3)	0.4
FAR^2^	15 (53.6)	59 (29.9)	0.01
Private insurance^2^	-	2(1)	0.5
Duration of evolution ¹ (months)	228.25± 101.25	210.75 ± 117.25	0.7
Diagnosis lag time^3^ (months)	14.50 (1 – 71.75)	12 (1 - 48)	0.9
Initiation Methotrexate lag time^3^ (months)	36 (3.12 – 80.98)	23.98 (0.13 – 60.03)	0.3
Sedimentation rate ¹ (mm/1^st^ hour)	41.42 ± 27.37	42.12 ± 24.46	0.5
C-reactive protein^3^ (mg/l)	19 (8 – 31.70)	22.50 (8 – 46.25)	0.3
DAS28 ¹	5.4 ± 1	5.2 ± 1	0.6
Positive rheumatoid factor * ^2^	23 (82.1)	169 (85.8)	0.3
Positive ACPA* ^2^	17 (60.7)	134 (68)	0.5
Carpite / erosions ^2^	14 (50)	97 (49.2)	0.2
Concomitant cDMARDs^2^	25 (89.3)	182 (92.4)	0.5
Arterial hypertension^2^	7 (25)	30 (15.2)	0.4
Diabetes^2^	8 (28.6)	38 (19.3)	0.4
Hypercholesterolemia^2^	5 (17.9)	23 (11.7)	0.3

1: Mean and standard deviation, 2: Number and percentage, 3: Inter-quartile median, * Data available ACPA: anti-citrullinated protein antibodies, RAMED (Régime d'Assistance Médicale aux économiquement démunis): health insurance for poor and vulnerable population, CNSS (Caisse Nationale de Sécurité Sociale): insurance for private sector employees, CNOPS (Caisse Nationale des Organismes de Prévoyance Sociale): insurance for public sector employees, FAR (assurance forces armées royales): insurance for military employees.

Data regarding the comparison between Rituximab, TCZ and TNFi as the first bDMARDs is presented in the Table 5. It is worth-noting that more Rituximab patients have the RAMED type of insurance compared to TCZ and TNFi (p = 0.002). The type of insurance FAR was higher in the TNFi group (53.6%) compared to the TCZ group (41.9%) and the Rituximab group (28.2%); p = 0.01. TCZ was far more considerably prescribed as mono-therapy compared to TNFi and Rituximab (p < 0.0001).

**Table 5:** comparison of the features between Rituximab vs TCZ vs TNFi as first bDMARDs

Features	Rituximab (n=166)	TCZ (n=31)	TNFi (n=28)	p
Age¹ (years)	51.94 ± 11.41	51.29 ± 10.78	52.64 ± 12.05	0.9
Female sex^2^	146 (88)	28 (90.3)	23 (82.1)	0.6
**Type of health insurance**				
RAMED^2^	81 (49.9)	12 (38.7)	4 (14.3)	0.002
CNSS^2^	3 (1.8)	1 (3.2)	1 (3.6)	0.7
CNOPS^2^	31 (19)	5 (16.1)	7 (25)	0.6
FAR^2^	46 (28.2)	13 (41.9)	15 (53.6)	0.01
Private insurance^2^	2 (1.2)	-	-	0.6
Duration of evolution¹ (months)	236 ± 132	150.33 ± 15.10	228.25 ± 101.46	0.3
Diagnosis lag time^3^ (months)	12 (1 - 48)	18 (1 - 49.5)	14.5 (1 - 71.75)	0.8
Initiation Methotrexate lag time^3^ (months)	18.10 (0.05-51.31)	53.99 (4.4-110.45)	36 (3.12-80.98)	0.3
Sedimentation rate ¹ (mm/1^st^ hour)	42.05 ± 24.30	42.5 ± 25.77	41.42 ± 27.37	0.9
C-reactive protein^3^ (mg/l)	24.5 (11.25 - 48.75)	9.5 (4.5 - 49)	19 (8 - 31.75)	0.2
DAS28 ¹	5.2 ± 1	5.1 ± 1	5.4 ± 1	0.6
Positive rheumatoid factor*^2^	144 (92.9)	25 (83.3)	23 (85.2)	0.1
Positive ACPA*^2^	115 (89.8)	19 (86.4)	17 (85)	0.7
Carpite/erosions^2^	82 (49.4)	15 (48.4)	14 (50)	0.4
Concomitant cDMARDs^2^	161 (97)	21 (67.7)	25 (89.3)	<0.0001
Arterial hypertension^2^	24 (14.5)	6 (19.4)	7 (25)	0.07
Diabetes^2^	34 (20.5)	4 (12.9)	8 (28.6)	0.08
Hypercholesterolemia^2^	18 (10.8)	5 (16.7)	5 (17.9)	0.4

1: Mean and standard deviation, 2: Number and percentage, 3: Inter-quartile median, * Data available ACPA : anti-citrullinated protein antibodies, RAMED (Régime d'Assistance Médicale aux économiquement démunis): health insurance for poor and vulnerable population, CNSS (Caisse Nationale de Sécurité Sociale): insurance for private sector employees, CNOPS (Caisse Nationale des Organismes de Prévoyance Sociale): insurance for public sector employees, FAR (assurance forces armées royales): insurance for military employees

In a multivariate analysis, the factors that remain associated with the choice of Rituximab as the first bDMARDs are the ones with the RAMED type of insurance OR = 2.5 (1.24-5.25); p = 0.01 and the concomitant intake of cDMARDs OR = 7.3 (2.35-22.79); p = 0.001 after an adjustment of age and a positive rheumatoid factor (Table 6).

**Table 6:** factors associated with the choice of Rituximab as the first bDMARDs in multivariate analysis

Features	Rituximab (n=166)		
	OR	IC (95%)	p
Age	1.02	(0.99-1.05)	0.1
Positive rheumatoid factor	2.5	(0.90-6.97)	0.07
Type of health insurance RAMED	2.5	(1.24-5.25)	0.01
Concomitant cDMARDs	7.3	(2.35-22.79)	0.001

## Discussion

The use of bDMARDs in controlling patients with rheumatoid arthritis has revolutionized the evolution of the disease [13]. Despite the broad spectrum and the constant development of biological treatment, there is no solid proof for the strategy of choosing the first bDMARDs. In the absence of determining factors, any bDMARDs validated for first line RA treatment can be used [8]. To the best of our knowledge, this is the first study showing the use of Rituximab as the first bDMARDs in the treatment of RA with a frequency of 166 (74%) patients, followed by TCZ 31 (13.6%) patients and then TNFi in the 3rd position 28 (12,4%) patients. In the Italian LORHEN registry [13] covering 1910 patients among which 1264 patients were treated by first line bDMARDs (TNFi first line: 1019, TCZ first line: 130, Abatacept first line: 115 patients). In a Brazilian study [15], 94 patients were collected of which 85 (90.4%) patients had received TNFi as first choice of bDMARDs, in second rank come Abatacept as first choice therapy then TCZ in four (4%) patients [15]. In the Swedish registry, Frisell T *et al*. [7] included 6481 patients who initiated a first line bDMARDs, most patients started TNFi (n = 5307, 82%), in the non-TNFi group Rituximab was the more prescribed as first biologic (n = 655, 10% of all first bDMARD) followed by Abatacept (n = 274) then TCZ (n = 245).

The international guidelines do not recommend the use of Rituximab as the first line of bDMARDs. However, in our study, that was the case and this can be explained by factors that influenced this choice, including the medico-economic factor and the risk of tuberculosis given the endemic context of this pathology in our country. Comparison of Rituximab vs. Non-Rituximab groups demonstrated that Rituximab initiators were dominantly RF-positive, they had a concomitant intake of cDMARDs and had the RAMED type of insurance: that is issued to patients who have a lower socio-economic level and who are absolved from medical treatment fees at the hospital. Rituximab is the most available biological drugs compared to other bDMARDs and it depends on the availability of the treatment. Frisell *et al*. [7] showed that patients who underwent Rituximab were old, less educated, had longer disease duration, more often seropositive and slightly higher ESR.

Several studies confirmed the evidence of the comparable efficacy of TCZ when prescribed as mono-therapy or in combination with cDMARDs [11, 12, 16, 17]. Our study confirms the significantly elevated prevalence of TCZ prescribing mono-therapy compared to Rituximab and TNFi. Gabay *et al*. [18] had a critical analysis of this approach; they mentioned that TCZ retention was shorter when prescribed as mono-therapy despite the comparable clinical response.

In our study, TCZ was also associated with a shorter duration of disease progression than the non-TCZ group. Concerning comorbidities, arterial hypertension was more represented in TCZ group compared to non-TCZ group and the opposite for diabetes that was associated with the non-TCZ group. Monti S *et al*. [13] revealed that age and comorbidities influence the choice towards abatacept (ABA) and TCZ compared to TNFi. Previous studies indicated that age and the presence of comorbidities are associated with a decreased response by Etanercept [19, 20]. Frisell T *et al*. [7] reported that patients receiving TNFi were younger than the non-TNFi group. In the literature, despite high levels of activity, the chances of receiving TNFi decrease with advancing age compared to younger patients [21, 22]. In our RA population, patients on TNFi had more FAR type of insurance than non-TNFi. No difference in age, disease activity, presence of hypertension or diabetes between TNFi and non-TNFi groups.

The limitations of our study relate to the small sample and the retrospective nature of the study which may lead to a partiality in the treatment as well as the selection of patients. Given the nature of the register, some data may be missed as a positive ACPA/RF example and therefore, their role in choosing the first line of bDMARDs cannot be assessed accurately and appropriately. Another limitation of our study relates to medicine´s costs and patients' preferences pertaining to the administrative routes. These costs and preferences could not be evaluated to guide treatment decisions, as this aspect is beyond our study design.

## Conclusion

In the literature, there is no clear consensus or international recommendations on the proper approach to prescribing the first biological treatment. This study, which was based on data from the Moroccan register of biotherapy patients with RA, suggests that there are different factors influencing the choice of the first line of bDMARDs in patients with RA. Rituximab was the most prescribed biological therapy as first line bDMARDS in our Registry; the insurance type influenced this choice. We conclude that the choice of Rituximab and TNFi is influenced by the type of health coverage and thus indirectly by the availability and the medico-economic cost of the biological.

### What is known about this topic

- Several studies have identified different factors influencing the choice of the first line of bDMARDs in patients with RA;
- Our study confirmed that Tocilizumab represents the best choice if mono-therapy is considered in rhumatoid arthritis.

### What this study adds

- This study presents divers strengths. It is a multicenter study of the first Moroccan and African register of biotherapies where Rituximab is used as the first line of bDMARDs given the context of endemic tuberculosis, the type of health insurance as well as the medical and economic cost of biologics;
- Other African registries with the same tuberculosis endemic context and different health coverage models are needed.
